# A prospective multicenter assessor-blinded randomized controlled study to compare the efficacy of short versus long protocols of electroconvulsive therapy as an augmentation strategy to clozapine in patients with ultra-resistant schizophrenia (SURECT study)

**DOI:** 10.1186/s13063-021-05227-3

**Published:** 2021-04-15

**Authors:** Virginie Moulier, Mohamed Wassim Krir, Marine Dalmont, SURECT Group, Bilal Bendib, Caroline Berjamin, Chérifa Benosman, Emeline Duhamel, Pierre Quesada, Benjamin Letertre, Jacques Bénichou, Vincent Compere, Clélia Quiles, Anne Sauvaget, Samuel Bulteau, Jean-Marie Vanelle, Edouard Laforgue, Antoine Yrondi, Etienne Véry, Marie Sporer, Christophe Arbus, Sonia Dollfus, Pierrick Lebain, Nemat Jaafari, Marie-Odile Krebs, Marion Plaze, Dominique Drapier, Patrick Le Bihan, Caroline Dubertret, Jérôme Attal, Pierre-Michel Llorca, Louis Foures, Dominique Januel, Noomane Bouaziz, Olivier Guillin, Maud Rothärmel

**Affiliations:** 1 University Department of Psychiatry, Centre d’Excellence Thérapeutique- Institut de Psychiatrie, Centre Hospitalier du Rouvray, Sotteville-lès-Rouen, France; 2 EPS Ville Evrard, Unité de Recherche Clinique, Neuilly-sur-Marne, France; 3 Faculté de Médecine, Normandy University, Rouen, France; 4 Rouen University Hospital, Rouen, France; 5 INSERM U 1245, University of Rouen, Rouen, France

**Keywords:** Electroconvulsive therapy, Ultra-resistant schizophrenia, Clozapine, Augmentation strategy, Randomized controlled trial

## Abstract

**Background:**

Although clozapine is the most effective antipsychotic drug for treatment-resistant schizophrenia, it leads to a poor or partial response in 40 to 70% of patients. Augmentation of clozapine with electroconvulsive therapy (ECT) is a highly effective and relatively safe treatment for these clozapine-resistant patients. However, parameters are not yet well specified, such as the optimal number of sessions, their frequency, and the relevance of maintenance ECT. Our objective is to compare the efficacy and tolerance between two protocols of combined ECT and clozapine treatment in patients with ultra-resistant schizophrenia (URS): a 6-month protocol (short protocol with 20 ECT sessions) and a 12-month protocol (long protocol with 40 ECT sessions).

**Methods:**

Sixty-four patients with schizophrenia with persistent psychotic symptoms despite clozapine treatment will be enrolled in a prospective multicentric assessor-blinded randomized controlled trial. Patients will be randomly assigned to the short or the long protocol. The main outcome is the response rate assessed by the Positive and Negative Symptoms Scale (PANSS) 3 months after the end of the treatment in patients following the long protocol compared to those following the short protocol. The response was defined as a 30% reduction on the PANSS baseline. Clinical assessments (PANSS, BPRS, HAMD-21, YMRS, CGI, GAF, Modified Overt Aggression Scale (MOAS), and Subjective Scale to Investigate Cognition in Schizophrenia (SSTICS)) and plasma clozapine concentration will be performed at baseline and at 2, 4, 6, 9, 12, and 15 months. Neuropsychological measures (MMSE, RL/RI-16, Doors test, D2 Test of Attention, Copy of the Rey-Osterrieth complex figure) will be performed at baseline and at 6 and 15 months.

**Discussion:**

The aims of this research are to optimize protocols of combined ECT with clozapine in patients with URS and to offer specific recommendations for these patients’ care.

**Trial registration:**

ClinicalTrials.gov NCT03542903. Registered on May 31, 2018. Id RCB: 2017-A02657-46

## Administrative information

**Table:** 

Title {1}	A prospective multicentric assessor-blinded randomized controlled study to compare the efficacy of short versus long electroconvulsive therapy protocols as an augmentation strategy to clozapine in patients with ultra-resistant schizophrenia (SURECT study)
Trial registration {2a and 2b}.	ClinicalTrials.gov: NCT03542903, registered on May 31, 2018. Id RCB: 2017-A02657-46.
Protocol version {3}	Protocol version number 5 (June 2, 2020)
Funding {4}	The study was financed by the French Ministry of Health (PHRCN 16-0401). The study’s promotor is the Centre Hospitalier du Rouvray.
Author details {5a}	a. University Department of Psychiatry, Centre d’Excellence Thérapeutique- Institut de Psychiatrie, Centre Hospitalier du Rouvray, Sotteville-lès-Rouen b. EPS Ville Evrard, Unité de Recherche Clinique, Neuilly-sur-Marne, France c. Faculté de Médecine, Normandy University, Rouen, France d. Rouen University Hospital, Rouen, France e. INSERM U 1245 University of Rouen, France
Name and contact information for the trial sponsor {5b}	The trial sponsor is the French ministry of Health through the Direction Générale de l’Offre de Soins (DGOS). Through a national clinical research project, the ministry supports projects that contribute to medical progress - whether technical or economical - as well as the improvement of practices and the quality of care. Direction Générale de l’Offre de Soins (DGOS) Bureau de l’innovation et de la recherche clinique 14 avenue Duquesne - 75350 Paris 07 SP mail: DGOS-PF4@sante.gouv.fr
Role of sponsor {5c}	Sponsor has only a financial role. It has no authority on analysis, interpretation and writing of the report.

## Introduction

### Background and rationale {6a}

Treatment-resistant schizophrenia (TRS) is a severe disorder with little to no response to antipsychotic drugs, affecting about 10 to 30% of patients with schizophrenia [1]. The lack of response to antipsychotics is associated in these patients with the worst community functioning among severely ill highly disabling psychiatric disorders, i.e., TRS, schizophrenia responsive to antipsychotics, bipolar disorder, anxiety/depressive diseases [2]. Suffering from persistent psychotic symptoms and cognitive dysfunctions, these patients with TRS manifested the poorest functioning in everyday life, being for the great majority unemployed and having not their own home [2]. Although clozapine is known to be the most effective antipsychotic drug in those cases, it leads to a poor or partial response in 40 to 70% of patients [3], and these patients are considered to have “ultra-resistant schizophrenia” (URS).

According to a recent meta-analysis of 18 randomized controlled trials comprising 1769 patients, ECT augmentation of clozapine had superior efficacy to clozapine as a monotherapy, was safe and relatively well tolerated by clozapine-resistant patients [4]. However, 17 of the 18 studies included in the meta-analysis were in Chinese, not allowing us to judge their scientific quality. Only one methodologically sound study was published in English by Petrides et al. [5]. In this prospective randomized study, 39 patients with clozapine-resistant schizophrenia were assigned either in the “ECT plus clozapine” group (*n* = 20) or in the “clozapine” group (*n* = 19). In the intent-to-treat analysis, ten of the 20 patients (50%) in the ECT augmentation group but none of the patients (0%) in the clozapine group met the response criterion. The response was defined as a 40% reduction in symptoms based on the psychotic symptom subscale of the Brief Psychiatric Rating Scale (BPRS), a Clinical Global Impressions (CGI)-severity rating of mild or less (less than 3), and a CGI improvement rating of much improved (less than or equal to 2). Augmentation of clozapine with ECT is therefore an extremely promising therapeutic strategy for patients suffering from URS, but there is a lack of randomized controlled trials investigating important methodological aspects such as the optimal number and frequency of sessions as well as the need for maintenance ECT. For example, it seems that a larger number of ECT sessions is required in patients with TRS than in depression [6], but without real experimental evidence. In fact, the duration of most studies about ECT in URS patients is relatively short (between 4 and 6 months), and a high relapse rate was reported in the weeks to months after ECT cessation [7]. In order to decrease the risk of relapse, some experts empirically recommended ECT protocols with a duration ranging from 6 to 12 months. To our knowledge, advantages and disadvantages of a short versus a long treatment protocol have never been investigated. In this context, we designed a prospective randomized controlled trial in order to compare the effects of two different duration protocols of combining ECT with clozapine in URS patients.

### Objectives {7}

Our main objective is to compare the efficacy between two combined ECT-clozapine protocols in patients with ultra-resistant schizophrenia (URS): a 6-month protocol (short protocol with 20 ECT sessions) and a 12-month protocol (long protocol with 40 ECT sessions).

We hypothesize that the long protocol will be more effective than the short protocol 3 months after the end of the treatment.

In the second aim, we will compare the effects of both protocols on clinical symptomatology throughout the study. We hypothesize that the clinical improvement will be superior in the long protocol than in the short protocol.

Finally, we will compare the impact of both protocols on cognition. We hypothesize that the cognitive side effects will be similar between the two groups, except for attention, the improvement of which would be greater in the long protocol. We assume indeed that the greatest clinical improvement expected in the long protocol can induce an attention enhancement.

### Trial design {8}

This study is a prospective, multicentric, randomized, two-arm, assessor-blinded trial.

Due to the severity of the patients’ condition, it is not ethically possible to conduct sham ECT sessions, making the blinding about the short or long arm impossible to maintain in patients. In this superiority trial, patients will be randomly assigned to one of both arms with an allocation ratio of 1:1.

## Methods: participants, interventions, and outcomes

The protocol study follows the SPIRIT recommendations.

### Study setting {9}

This trial will be conducted by the Research Department of the Centre Hospitalier du Rouvray (Sotteville-lès-Rouen, France) in collaboration with 13 French clinical centers (Table 1).

**Table 1:** List of the centers and investigators (SURECT group)

Investigators	Center/department
Dr. Maud Rothärmel	Centre Hospitalier du Rouvray, Sotteville-lès-Rouen
Pr. Olivier Guillin	Centre Hospitalier du Rouvray, Sotteville-lès-Rouen
Pr. Jacques Bénichou	Rouen University Hospital, Rouen
Pr. Vincent Compere	Rouen University Hospital, Rouen
Dr. Clélia Quiles	Centre Hospitalier Charles Perrens, Bordeaux
Pr. Anne Sauvaget	Nantes University Hospital, Nantes
Dr. Samuel Bulteau	Nantes University Hospital, Nantes
Pr. Jean-Marie Vanelle	Nantes University Hospital, Nantes
Dr. Edouard Laforgue	Nantes University Hospital, Nantes
Dr. Antoine Yrondi	Toulouse University Hospital, Toulouse
Dr. Marie Sporer	Toulouse University Hospital, Toulouse
Dr. Christophe Arbus	Toulouse University Hospital, Toulouse
Pr. Sonia Dollfus	Caen University Hospital, Caen
Dr. Pierrick Lebain	Caen University Hospital, Caen
Pr. Nemat Jaafari	Centre Hospitalier Henri Laborit, Poitiers
Pr. Marie-Odile Krebs	Centre Hospitalier Saint Anne, Paris
Dr. Marion Plaze	Centre Hospitalier Saint Anne, Paris
Pr. Dominique Drapier	Centre Hospitalier Guillaume-Reignier, Rennes
Dr. Patrick Le Bihan	Centre Hospitalier de Cadillac, Cadillac
Pr. Caroline Dubertret	Louis Mourier Hospital, Colombes
Dr. Jérôme Attal	Montpellier University Hospital, Montpellier
Pr. Pierre-Michel Llorca	Clermont-Ferrand Hospital, Clermont-Ferrand
Pr. Dominique Januel	EPS Ville Evrard, Neuilly-sur-Marne
Dr. Noomane Bouaziz	EPS Ville Evrard, Neuilly-sur-Marne

### Eligibility criteria {10}

Patients eligible for the study are patients with schizophrenia continuing to experience persistent psychotic symptoms despite a well-managed clozapine treatment.

#### Inclusion criteria

- Patients aged 18 to 55
- Patients with schizophrenia as defined by the DSM-5 criteria
- Patients had at least two previous unsuccessful treatment trials with conventional or atypical antipsychotics from two different classes at a dose of ≥600 mg chlorpromazine equivalent
- Patients had received clozapine for at least 6 weeks prior, with a plasma concentration ≥ 350 ng/ml
- Patients continuing to experience persistent positive psychotic symptoms with a score of at least 4 (moderate) on at least two of the four positive symptoms of the BPRS (grandiosity, suspiciousness, hallucinations, and unusual thoughts)
- Current presence of at least moderately severe illness, with total BPRS score ≥ 45 and a CGI-S score ≥ 4 (moderate)
- Patients had a stable treatment for at least 8 weeks (antipsychotics, mood stabilizers, and antidepressants)
- Patients affiliated to a social security system
- Patients able to understand both spoken and written French
- Patients giving their informed written consent and agreement of their legal guardian for patients under guardianship
- Patients deprived of liberty if they gave their informed, written consent

#### Non-inclusion criteria

- Patients with current depressive, manic, or hypomanic episodes as defined by the DSM-5 criteria
- Patients who had ECT during the last 6 months
- Patients with unstable epilepsy
- Patients with a severe neurological or systemic disorder that could significantly affect cognition, behavior, or mental status (other than tardive dyskinesia or neuroleptic-induced parkinsonism)
- Patients with one or more of the following conditions (in order to minimize risks related to ECT and general anesthesia): intracranial hypertension, recent myocardial infarction, advanced coronary failure, embolic disease, recent stroke, the existence of expansive intracranial lesions without intracranial hypertension, the presence of aneurysms or cerebral vascular malformations with bleeding risk, aneurysm of the aorta, the existence of a retinal detachment, the existence of a pheochromocytoma, and serious heart or respiratory failure
- Patients with a severe substance use disorder (other than nicotine or caffeine) according to the DSM-5 criteria
- Patients with concomitant use of benzodiazepines and antiepileptic drugs, except for lamotrigine
- Pregnant or lactating women and women of childbearing age without adequate contraception
- Patients with contraindications to etomidate or any of its excipients and/or neuromuscular blocking agents
- Patients participating, or having participated within the 30 days prior to the inclusion visit, in an interventional clinical trial

#### Exit criteria

- The patients are definitively excluded from the study in the following cases: removal of the patient’s consent or the legal guardian agreement (in these cases, the collected data are not included in the data analysis)
- Patients refusing to continue the study, without removal of the consent (in these cases, the collected data will be included in the data analysis)
- Decision of the investigator: worsened clinical condition, occurrence of significant adverse effects, need to change the background drug treatment, and impossibility for the patient to follow the established protocol
- Death of the patient;
- Patient lost to follow-up (after unsuccessfully trying to contact the patient three times at 1-week intervals)

### Who will take informed consent? {26a}

The potential patients will receive complete and adequate information about the protocol, and they will have a reflection period of at least 7 days before the signature of the informed consent in the presence of the investigators. If patients are under legal guardianship, written agreement from their legal guardians should also be obtained.

### Additional consent provisions for collection and use of participant data and biological specimens {26b}

On the consent form, participants will be asked if they agree to use of their data should they choose to withdraw from the trial. Participants will also be asked for permission for the research team to share relevant data with people from the universities taking part in the research or from regulatory authorities, where relevant. This trial does not involve collecting biological specimens for storage.

### Interventions

#### Explanation for the choice of comparators {6b}

Patients will be randomized to receive a short ECT protocol or a long ECT protocol.

##### Short ECT protocol

The short ECT protocol is a standard protocol to treat URS patients, inspired by the continuation protocols commonly used in depression [8]. However, it is limited by a relatively high relapse rate. In this protocol, 20 sessions of ECT will be administered in 6 months with the following schedule: twice a week for 6 weeks and then once a week for 4 weeks. Afterwards, the patients will have ECT sessions every 3 weeks for 6 weeks and then each month for 2 months.

##### Long ECT protocol

Increasing the number of sessions, increasing the duration of treatment, and reducing the frequency more gradually could decrease the risk of relapse in URS patients.

In this long protocol, all the phases are doubled and could resemble a maintenance protocol used in depression [9]. In the long arm, 40 sessions of ECT will be administered in 12 months with the following schedule: twice a week for 12 weeks and then once a week for 8 weeks. Afterwards, the patients will have ECT sessions every 3 weeks for 12 weeks and then each month for 4 months.

### Intervention description {11a}

#### ECT equipment

For the two protocols (short and long), the main ECT devices which will be used are the THYMATRON System IV (SOMATICS, LLC, 720 Commerce Drive Venice, Floride 34292, USA) or the spECTrum 5000Q (MECTA Company/Micromed, 7015 SW McEwan Rd., Lac Oswego, OR 97035, USA). ECT will be administered through bilaterally positioned electrodes on the temporal region. The stimulation dose will be determined by the titration method during the first ECT session [10].

The dose for therapeutic stimulation will be twice the seizure threshold. As recommended, this dose may be increased if the seizure does not meet the effectiveness criteria.

#### Anesthesia procedure

The patient should have an anesthesia assessment during the month prior to ECT onset. An interview with the anesthetist should also be conducted prior to each ECT session. The patient should not eat or drink anything for the 12 h leading up to each session, except for cases of premedication. The administered pharmacological agents are written down on the anesthesia sheet. Infusion therapy is provided. Blood pressure, heart rate, and blood saturation of oxygen will be monitored. An adapted emergency box with all drugs needed to control any cardiovascular effects will be at disposal and checked before each session.

Loss of consciousness is insured by the administration of one short-lasting hypnotic drug: etomidate (between 0.1 and 0.7 mg/kg). This dose should allow recovery within 5 to 10 min. Patient safety is guaranteed by the administration of a short-lived curare: suxamethonium chloride (between 0.8 and 1.2 mg/kg), a dose allowing both the decrease of the muscular contraction intensity and spontaneous breathing recovery within 3 or 5 min. The required doses are adapted to each patient by the anesthetist. During each ECT session, the anesthetist records cardiovascular parameters (heart rate and systolic and diastolic blood pressure). After the convulsive phase, breathing enriched with oxygen is insured by mask ventilation until spontaneous breathing recovery, after which the patients remain 1 h in the recovery room. Then, they are taken by ambulance back to their wards. Getting back to normal eating habits happens 1 h and a half to 2 h after recovery.

The medical team to be present during the ECT session is composed of the psychiatrist who is responsible for administering the electroshock, the anesthetist, and the nurse anesthetist.

#### Criteria for discontinuing or modifying allocated interventions {11b}

The patients stop participating in the study in the following cases:

- Removal of the patient consent or the legal guardian agreement (in these cases, the collected data are not included in the data analysis)
- Patients refusing to continue the study, without removal of consent (patients’ data may be analyzed)
- Decision of the investigator: if the patient’s clinical condition worsens, significant adverse effects occur, wish of the patient to receive more ECT when he/she is assigned to the short arm, need to change the background drug treatment, impossibility for the patient to follow the established protocol
- Death of the patient;
- Patients lost to follow-up (after unsuccessfully trying to contact the patient three times at 1-week intervals)

### Strategies to improve adherence to interventions {11c}

At each visit, the plasma clozapine concentration is measured to check treatment compliance.

### Relevant concomitant care permitted or prohibited during the trial {11d}

The patients continue their usual medical treatments prescribed by their psychiatrists or their general practitioners. Concurrent use of antipsychotic medication, antidepressants, and lithium is allowed as long as the dose is stable for at least 8 weeks before entering the study. Loxapine (up to 300 mg per day) or hydroxyzine (up to 100 mg per day) may be used to treat anxiety, agitation, or insomnia. Once randomly assigned to one of the groups, the same clozapine dose will be kept throughout the study. The lithium dosage may be adjusted to obtain low levels of lithemia and thus promote tolerance of treatment during ECT. However, patients should not be treated by another electrical or magnetic stimulation method. Anticonvulsant drugs must be stopped (antiepileptics and benzodiazepines) except for lamotrigine. Indeed, lamotrigine is not associated with a decrease in ECT efficacy [11].

### Provisions for post-trial care {30}

No ancillary study is planned for this protocol. At the request of the patients or their psychiatrists, the patients could benefit from new ECT sessions at the end of the protocol.

### Outcomes {12}

#### Main outcome

The main outcome is the response rate assessed by the Positive and Negative Symptoms Scale (PANSS) 3 months after the end of the treatment in patients following the long protocol (i.e., at 15 months) compared to those following the short protocol (i.e., at 9 months), the response being defined as a 30% reduction on the PANSS [12].

#### Secondary outcomes

The secondary outcomes are as follows:

- The response rate assessed by the Brief Psychiatric Rating Scale-18 items (BPRS-18) 3 months after the end of the treatment in patients following the long protocol compared to those following the short protocol (the response being defined as a reduction of 30% on the BPRS).
- The response rates assessed by the PANSS and the BPRS at different times (2, 4, 6, and 12 months) between both groups.
- The Hamilton Rating Scale-21 items (HAMD-21) score, the Young Mania Rating Scale (YMRS) score, the Modified Overt Aggression Scale (MOAS) score, the Clinical Global Impression (CGI) score, and the Global Assessment Functioning (GAF) score at baseline and at 2, 4, 6, 9, 12, and 15 months. Changes from baseline will be compared between both groups.
- The Mini-Mental Status Examination (MMSE), the Subjective Scale to Investigate Cognition in Schizophrenia (SSTICS) [13], the RL/RI-16 test assessing verbal memory [14], the Doors test assessing visual memory [15], the D2 Attention Test [16], and the Copy of the Rey-Osterrieth Complex figure test for the evaluation of visuospatial constructional ability [17] at baseline and at 6 and 15 months. Changes between baseline and 6 months and between baseline and 15 months will be compared between the groups.
- Plasma clozapine concentration measured at baseline and at 2, 4, 6, 9, 12, and 15 months in order to check patient compliance.

### Participant timeline {13}

**Fig. 1:**
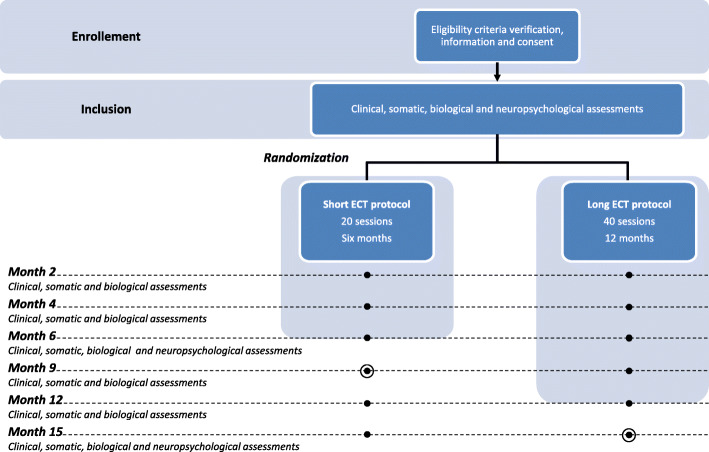
Flow chart of the study design. The main objective was the comparison of the response rate 3 months after the end of each treatment (black circles surrounded)

**Fig. 2:**
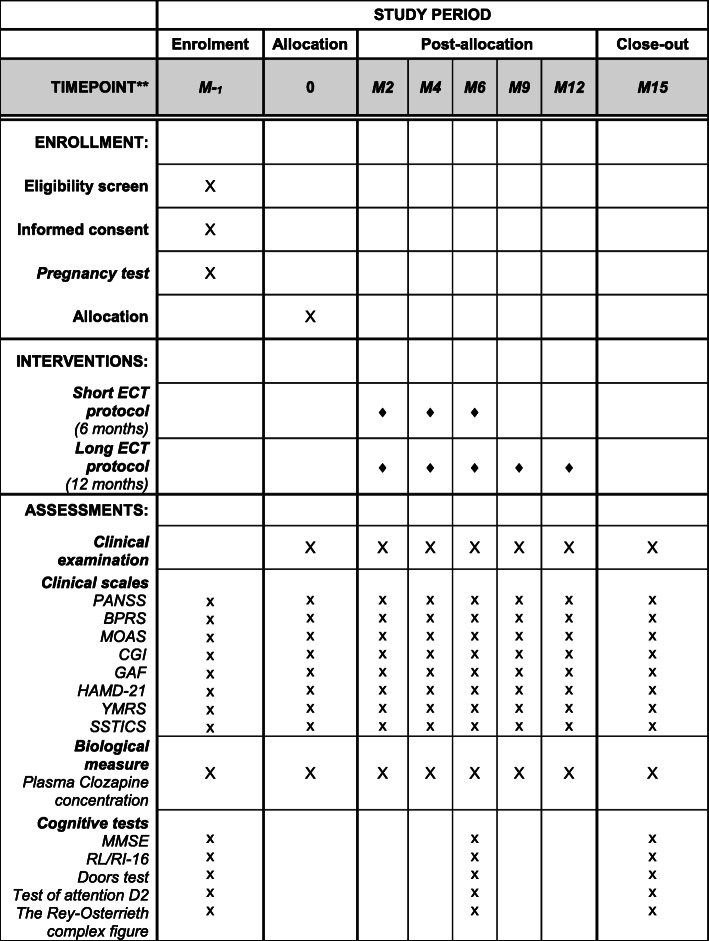
Schedule of enrollment, interventions, and assessments of the study. PANSS, Positive and Negative Symptoms Scale; BPRS, Brief Psychiatric Rating Scale-18 items; MOAS, Modified Overt Aggression Scale; CGI, Clinical Global Impression; GAF, Global Assessment Functioning; HAMD-21, Hamilton Rating Scale-21 items; YMRS, the Young Mania Rating Scale; SSTICS, Subjective Scale to Investigate Cognition in Schizophrenia; MMSE, Mini-Mental Status Examination

Experimental design, the time of interventions, and assessments are presented in Figs. 1 and 2.

### Sample size {14}

The aim of this trial is to compare the response rates between short and long ECT protocols. According to the meta-analysis of Lally et al. [7], the proportion of patients with treatment-resistant schizophrenia that responds to ECT augmentation of clozapine was 61.79% (Table 1: 55/89 cases). However, the relapse following ECT cessation is very high: the relapse rate has been estimated at 62.5% by Kho et al. [18]. On the other hand, Braga et al. [19] suggested that ECT were effective as a continuation strategy to prevent relapse after an acute course of ECT. During the 6-month maintenance period, no patient presented with clinically worsening symptoms. From this literature data, we postulate that the responder rates would be 61.79% in the long ECT protocol and 23.25% in the short ECT protocol (62% × (1 − 0.625) = 23.25%). Assuming an alpha risk of 0.05 and a power of 0.80, we estimated the need to include 25 patients per group. To overcome an estimated 20% drop-out, the required number of patients increased to 32 patients per group. As a result, the total number of participants required for the study is 64 patients.

### Recruitment {15}

Enrollment will occur in 13 clinical centers (Table 1) during 48 months: each center should include 1.2 patients per year, which is far below the usual clinical activity of these centers.

## Assignment of interventions: allocation

### Sequence generation {16a}

Web-based randomization will assign patients to benefit from short or long ECT protocol in a 1:1 ratio with balanced blocks thanks to the Clinsight software. Block size is unknown to investigators. Stratification according to the clinical center will be performed. A file describing the random procedure is confidentially classified within the Biostatistics Unit in the University Hospital of Rouen.

### Concealment mechanism {16b}

On demand, the informed investigator will obtain the patient number and the arm in which the latter has been assigned on the Clinsight website.

### Implementation {16c}

The Biostatistics Unit in the University Hospital of Rouen generated the allocation sequence, which has been downloaded on the Clinsight website. Participants will be enrolled by the investigators of each clinical center (Table 1). However, only informed non-blind investigators will assign participants to an arm.

He then informs the research technician about the organization of the ECT protocol to perform. Among the investigators, the psychiatrist receiving the information of the treatment allocation is different from the psychiatrist who performs clinical assessments.

## Assignment of interventions: blinding

### Who will be blinded {17a}

The investigators, who clinically assessed the patients, and psychologists, who administered cognitive tests, are blinded. The data analysts will be blind until the entire analysis is completed. However, patients and care providers will be informed of the treatment allocation, due to the impossibility of maintaining the blindness in arms with different durations.

### Procedure for unblinding if needed {17b}

As this study is single-blind and an investigator is informed of the arm in each clinical center, the question of revealing the blindness does not arise.

## Data collection and management

### Plans for assessment and collection of outcomes {18a}

As the clinical scales used are part of the current medical practice and are passed by experienced psychiatrists, no training was necessary. The validity and reliability of scales are described in Table 2.

**Table 2:** Validity and reliability of the main and secondary outcomes

	Validity	Test-retest reliability and/or sensitivity to change	Inter-rater reliability
Main outcome			
Positive and Negative Syndrome Scale (PANSS) [12]	High internal consistency with an alpha coefficient comprised between 0.73 and 0.83	Reliable test-retest accuracy (0.77 to 0.89)	High correlations, comprised between 0.83 and 0.87
Secondary outcomes			
Brief Psychiatric Rating Scale (BPRS) [20]	Internal consistency: alpha = 0.46 for general score [21]	–	Inter-rater reliability = between 0.87 and 0.97 [22]
Young Mania Rating scale (YMRS) [23]	Concurrent validity: correlation between YMRS and Mania Assessment Scale was very high and statistically significant at each weekly assessment (*r* > 0.91, *p* < 0.001) [24]	Sensitivity to change because there was a statistically significant decline among 15 patients after 2 weeks of treatment [25]	Inter-rater reliability ICC > 0.89 [24]
Modified Overt Aggression Scale (MOAS) [26]	Convergent validity: *r* = 0.75 with history of actual aggressive behavior (*p* < 0.001) Divergent validity: *r* = − 0.09 with Eysenck Personality Questionnaire Extraversion [27]	ICC = 0.6 [28]	ICC = 0.96 [29]
Clinical Global Impression Scale (CGI)-Severity [30]	Positive correlation between CGI-S and HAM-D, anticipatory anxiety, and panic frequency in a sample of 116 patients with panic disorder and depression [31]	–	Inter-rater reliability = 0.66 in 12 patients with dementia [32]
Global Assessment of Functioning scale (GAF) [33]	The multiple regression between the GAF and the measures of symptoms and social behavior were large and highly significant (*r* = − 0.63 with the SANS total and *r* = − 0.46 with the Social Behavior Schedule [34]. A longer hospitalization was associated with lower baseline GAF (OR = 1.91) [35]	–	ICC = 0.89 to 0.95 [34]
Mini-Mental Status Examination (MMSE) [36]	Convergent validity: *r* = 0.7 to 0.9 with other cognitive screening tests [37]	Test-retest reliability coefficients = 0.80 to 0.95 [37]	Inter-rater reliability was found to be high (mean kappa value = 0.97) [38]
Subjective Scale to Investigate Cognition in Schizophrenia (SSTICS) [13]	Convergent validity: the SSTICS total score positively correlated with the Frankfurt-Pamplona Subjective Experiences Scale total score (*r* = .541, *p* < .01) [39] Good internal consistency*:* Cronbach’s alpha = 0.858 [13]	Test-retest coefficient: *r* = 0.82 (*p* < 0.01) for the two global scores [13]	–
RL/RI-16 [40]	RL/RI-16 is sensitive enough to differentiate patients with Alzheimer’s disease from those with mild cognitive impairment [41], vascular dementia [14] and fronto-temporal dementia [42].	Use of a parallel form whose performances are not different from the basic list except for free recall 2 and delayed free recall (parallel form > basic list)	–
Doors test [15]	Moderately strong correlations with visual recognition memory task (*r*^2^ ≅ 0.60) [43].	–	Inter-rater reliability: excellent agreement (*r* = .98) between two independent raters in a sample of 237 subjects [44]
Test of attention D2 [16]	Internal consistency: Cronbach’s alpha = 0.97 for total score [45].	The ICCs for the seven subscores of the D2 between successive sessions were between 0.78 and 0.94 [46]	
The Rey-Osterrieth complex figure [17]	–	Test-retest reliability coefficients = 0.60 to 0.76 [47].	The inter-rater reliability for direct copying scores was *r* = 0.96 (*p* < 0.0001) [48].

*ICC* intra-class correlation coefficient

### Plans to promote participant retention and complete follow-up {18b}

No specific strategy is planned to promote participant retention. However, resistant schizophrenic patients are mostly long-term hospitalized patients because of their serious illness. Being hospitalized can guarantee adherence to care. As far as possible, if the patient wishes to stop ECT sessions, it is recommended to proceed to the next follow-up visit in order to have the most concomitant data of the end of the ECT.

### Data management {19}

All required information in the protocol should be recorded on the case report forms with an explanation for any missing data. The data should be collected as soon as the information is obtained. The data should be clearly transcribed in these forms. The mistaken data will be corrected by the investigator or the person allowed to make the relevant correction.

As the data are collected on an electronic case report form (eCRF), the traceability of amendments and updates is automatic (audit-trail).

### Confidentiality {27}

All the data collected in the eCRF are anonymized, that is to say that the patient receives a code name according to their inclusion center and their randomization number. Only their initials and their month and year of birth are noted. All documentation pertaining to the study (protocol, consent, case report forms, investigator folder, etc.) as well as original documents (laboratory results, neuroimaging data, consultation reports, reports of clinical examinations, etc.) will be kept in a safe place and considered as confidential material. Data archiving will be the responsibility of the investigator and in accordance with the laws in place. The investigator will retain the data as well as the patient identification list for a minimum period of 15 years after the end of the study.

### Plans for collection, laboratory evaluation, and storage of biological specimens for genetic or molecular analysis in this trial/future use {33}

None of these procedures is planned in the study.

## Statistical methods

### Statistical methods for primary and secondary outcomes {20a}

Patient characteristics will be described overall and by randomly allocated treatment arm (short or long therapy) using the usual parameters, i.e., mean, standard deviation, median, interquartile range, and range for quantitative characteristics and frequencies for categorical characteristics.

The main outcome (the response rate in the PANSS three months after treatment cessation) being categorical, Pearson’s chi-square test will be used. The same strategy will be applied for the other categorical criteria (secondary outcomes). For the secondary outcomes with quantitative measures, the comparison between both groups will be performed thanks to the repeated measures ANOVA or Mann-Whitney tests based on delta scores (i.e., month 6 minus baseline, month 15 minus baseline), when the assumptions of variance equality and/or normal distribution are violated. If multiple Mann-Whitney tests are performed, Bonferroni adjustment will be applied to compensate for the risk of type 1 errors.

### Interim analysis {21b}

No interim analyses are planned.

### Methods for additional analysis (e.g., subgroup analyses) {20b}

This analysis will be complemented by a comparison using the logistic regression model in order to adjust for center and possible prognostic characteristics.

### Methods in analysis to handle protocol non-adherence and any statistical methods to handle missing data {20c}

Comparison of the two treatment arms will be based on the intent-to-treat principle. The maximum bias hypothesis shall be considered for non-assessed patients 3 months after the end of the treatment (patients lost to follow-up). Those patients, if in the group with the most favorable evolution, shall be considered as having not responded. On the contrary, those patients, if in the group with the most unfavorable evolution, shall be considered as responding to treatment. The aim is to reduce the gap between both to a minimum. By doing so, a robust conclusion is expected. Then, several sensitivity analyses shall be carried out: one analysis excluding patients lost to follow-up (per-protocol analysis) and another imputing the missing data by the last observed value (“last observation carried forward”).

### Plans to give access to the full protocol, participant level-data, and statistical code {31c}

The datasets analyzed during the current study are available from the corresponding author on reasonable request.

## Oversight and monitoring

### Composition of the coordinating center and trial steering committee {5d}

The research department of the Rouvray Hospital coordinates the trial. The department consists of three psychiatrists, a clinical research associate, a research engineer, and three research technicians.

### Composition of the data monitoring committee, its role, and reporting structure {21a}

The clinical research associate appointed by the promoter shall regularly visit the center in which the trial is led:

- At trial implementation
- During the trial
- At the end of the trial

The clinical research associate shall ensure that subjects’ fundamental rights and security are respected, as well as reliability, quality, and traceability of the transmitted data. The clinical research associate shall check that the study is led in compliance with the protocol, in good clinical practice, and within the legal regulatory framework.

The purpose of the visits is to validate the following:

- Eligibility of the patients involved: respect of inclusion/exclusion criteria
- Compliance with the procedures of information sharing with the patients and consent collection
- Compliance with the specific procedures of the protocol, trial schedule, and patient follow-up
- Quality of the collected data in the CRF (accurate, complete, and consistent)
- Compliance with the procedures reporting serious adverse event (SAE)
- Good management and traceability of the treatments/devices on trial (visit to the pharmacy, storing, and accounting of the drugs/devices)

At the end of each visit, a standardized monitoring report must be drafted by the clinical research associate. This report is reviewed by the promoter.

### Adverse event reporting and harms {22}

The investigator is responsible for assessing each adverse event in relation to its seriousness.

The investigator should record any adverse event in the AE section of the case report form (CRF).

Whenever possible, symptoms should be grouped as a single syndrome or diagnosis. The investigator should specify the date of onset, intensity, action taken, outcome of all adverse events, and their opinion as to whether the adverse effect can be related to the study. All events that meet one or more criteria of seriousness will be reported as serious adverse events.

In a case of a serious adverse event, the investigator should immediately (within 1 working day) do the following:

- Send the complete SAE form to the sponsor by fax:
  Ms. Ingrid Fontaine
  Centre Hospitalier du Rouvray
  Fax: +33 02 32 95 12 75
  All SAE forms should be dated and signed by the investigator.
- Specify the SAE diagnosis and describe the event and include the action taken. If the diagnosis is unknown at the time of the report, or if the diagnosis changes after investigations, a detailed written follow-up should be sent to the sponsor to provide the final diagnosis.
- Attach the copies of all examinations carried out and the dates on which these examinations were performed. Care should be taken to ensure that the patient’s identity is protected, and the patient’s identifiers in the clinical trial are properly mentioned on any copy of the source document. For laboratory results, it must include relevant negative results and the laboratory normal ranges.
- Joint report of hospitalization related to SAE.
- Assess the causality between the study procedures and SAE.
- Ensure that relevant information is communicated to the sponsor and when it becomes available.
- Monitor the patient with an SAE to its final resolution, stabilization at a level deemed acceptable by the investigator, or a return to the patient’s original state, even if the patient is no longer in the trial, and inform the sponsor of the SAE evolution.

### Frequency and plans for auditing trial conduct {23}

An audit can be realized at any time by people appointed by the promoter, the Centre Hospitalier du Rouvray. They are independent of the people in charge of the research. The objective is to ensure the quality of the research, the validity of its results, and the respect of the law. The investigators agree to conform to the requirements of the promoter and to the Competent Authority as regards an audit or an inspection from the trial. The audit can occur in all the stages of the study.

### Plans for communicating important protocol amendments to relevant parties (e.g., trial participants, ethical committees) {25}

Any modification to the protocol should be approved by the Independent Ethics Committee (art. L1123-6 of French Public Health Code) that authorized the start of the trial. Information is also made to the ANSM (French Competent Authority) (art. L 1123-8 of French Public Health Code).

If the modifications are accepted, the new version of the protocol is sent to all the investigative centers, and the modifications are applied. Changes will also be made on ClinicalTrials.gov.

### Dissemination plans {31a}

Analysis of the results will be communicated in conferences and scientific publications. Publication texts and communications will be discussed with all participating investigators. The order of the co-authors takes into account the participation of different investigators in the trial.

## Discussion

Even though ECT is recommended for patients with URS, there is a noticeable lack of rigorous studies on this subject. Parameters such as the frequency of ECT sessions, the position of electrode(s), the seizure efficacy criteria, or the anesthetic management have been little studied and should be tested. In addition, the utility of maintenance ECT in patients with URS has never been studied, except in open studies and clinical cases. The present study focuses on the optimal duration of ECT treatment, aiming to compare two protocols of combining ECT with clozapine: a 6-month protocol and a 12-month protocol.

The minimum number of ECT sessions to achieve significant clinical improvement in patients with URS ranges between 16 and 20 [49, 50]. In a recent meta-analysis about ECT augmentation of clozapine for clozapine-resistant schizophrenia, the mean duration of the included studies was only 8–9 weeks [4]. The authors suggested that longer-term follow-up data are needed in order to assess if patients maintain the clinical improvement that they achieved from ECT augmentation of clozapine, or whether maintenance ECT is required. The present study should try to answer this important question. We hypothesized that the long protocol will be more effective than the short protocol. It is important to accurately assess the efficiency gain because an increased duration of treatment leads to an additional cost and decreases the number of potentially treated patients. A long protocol could also allow a better stabilization of patients, avoid relapses and hospitalizations, and thus improve their cognitive profile and their quality of life.

Some methodological choices we made are worth discussing.

First, the duration of participation in this study is long (15 months in total). There is therefore a risk of having a significant number of patients lost to follow-up, especially in severely disabled patients. In the sample size calculation, the drop-out rate was estimated to 20% and the number of patients increased to overcome the reduction of statistical power. However, we lack data to accurately estimate this rate.

Secondly, the present study is a single-blind study because the treatment duration is different between both groups. Sham ECT, consisting in a brief anesthesia not followed by ECT, could theoretically allow blinding, but the confusion that often occurs post-ECT may unblind the arm [7]. In addition, the exposition of patients to the potential risk of anesthesia without active ECT sessions is not ethically acceptable.

Thirdly, the short protocol was established according to what is usually done in depression, considering a shock treatment phase (2 sessions per week) and a continuation phase (spacing of sessions to once a week for 4 weeks, then every 15 to 21 days for 2 months then monthly) [8]. As some authors have suggested that a longer course of treatment was necessary for schizophrenia [6], the duration of each of the phases (attack/shock and continuation phases) was doubled in the long protocol. We can question the relevance of these different durations which can be adjusted later.

Finally, we choose to assess cognitive functions because cognitive impairment is a frequent consequence of ECT. However, the extent of its persistence is debated [50]. Is it an acute, subacute, or persistent adverse effect? On the contrary, certain cognitive domains could progress because of the clinical improvement due to ECT. In a recent review, Ali et al. [51] highlighted the absence of evidence of persistent cognitive impairment in patients suffering from resistant schizophrenia after ECT. One month after completing ECT treatment, a full memory recovery in ten patients with schizophrenia was indeed reported [52]. Regarding memory, attention, and frontal functions, there was no difference between the ten patients with schizophrenia treated by maintenance ECT (mean duration of ECT = 13.5 months) and the ten patients matched for diagnosis, sex, and age and who had never been treated with ECT [53]. In a prospective open study, Vuksan Cusa et al. [54] investigated the effects of ECT augmentation on cognitive functions in 31 patients with treatment-resistant schizophrenia. After a mean number of 10.2 ECT sessions (range 7 to 14), the immediate and delayed total recall (measure of verbal memory) and the performance on the Stroop Interference Test (measure of executive functioning and cognitive flexibility) were improved, with no change on other cognitive measures. However, in this study, treatment consisted of acute ECT, and not maintenance ECT, and there was no control group. In maintenance ECT, the number of sessions increases significantly in comparison with acute ECT. For example, in a clinical series, Rothärmel et al. [55] indicated that two patients, with a particularly severe initial symptomatology and aggressive behavior, had maintenance ECT with a total of 50 and 60 sessions, over periods of 12 and 36 months, respectively. We ignore the cognitive effect of such a number of ECT sessions. Nevertheless, the authors point out that one of the patients had been able to leave the hospital after several years of hospitalization to find a place in society, thus suggesting better cognitive functioning.

## Perspectives

The results of this study should bring some answers about ECT as an augmentation strategy to clozapine, in order to improve the treatment of patients with URS. These questions relate to the number and frequency of ECT sessions to prevent the risk of relapse. They also concern the cognitive tolerance of ECT sessions and their effect on certain dimensions of schizophrenia such as positive symptoms but also aggressive behavior.

Other questions remain to be explored, such as the repercussions on patients’ brain imaging or the pathophysiological mechanisms underlying ECT.

## Conclusion

The current clinical trial will investigate the optimal duration, frequency, and number of ECT sessions combined with clozapine in patients with URS. Clinical and cognitive effects will be compared between two protocols: a 6-month protocol with 20 ECT sessions and a 12-month protocol with 40 ECT sessions, where each ECT phase is doubled. This study will provide new insight to treat patients with URS, optimizing the ECT procedure and limiting undesirable effects.

### Trial status

This is the fifth version of the protocol dated June 2, 2020. Recruitment started on July 4, 2018, and we anticipate recruitment will end on July 2022.

## Abbreviations

BPRS: Brief Psychiatric Rating Scale
CGI: Clinical Global Impressions
DGOS: Direction Générale de l’Offre de Soins
ECT: Electroconvulsive therapy
eCRF: Electronic case report form
GAF: Global Assessment Functioning
HAMD-21: Hamilton Rating Scale-21 items
MMSE: Mini-Mental Status Examination
MOAS: Modified Overt Aggression Scale
PANSS: Positive and Negative Symptoms Scale
SAE: Serious adverse event
SSTICS: Subjective Scale to Investigate Cognition in Schizophrenia
TRS: Treatment-resistant schizophrenia
URS: Ultra-resistant schizophrenia
YMRS: Young Mania Rating Scale
